# Supplementary material for: Effects of Multi-Task Mode on Cognition and Lower Limb Function in Frail Older Adults: A Systematic Search and Review
Source: Healthcare (Basel). 2023 Nov 21;11(23):3012. doi: 10.3390/healthcare11233012 (PMC10706791; doi:10.3390/healthcare11233012)
Supplement: Supplementary file 1 [file healthcare-11-03012-s001.zip › healthcare-2632179-supplementary.pdf]

**Supplementary File S1. Search strategy for PubMed**

| No. | Search terms |
|---|---|
| #1 | dual-task [MeSH Terms] |
| #2 | multi-task [MeSH Terms] |
| #3 | dual-task [Title/Abstract] |
| #4 | multi-task [Title/Abstract] |
| #5 | #1 OR #2 OR #3 OR #4 |
| #6 | limb function [MeSH Terms] |
| #7 | muscle function [MeSH Terms] |
| #8 | motor function [MeSH Terms] |
| #9 | exercise function [MeSH Terms] |
| #10 | balance [MeSH Terms] |
| #11 | postural control [MeSH Terms] |
| #12 | balance control [MeSH Terms] |
| #13 | physical performance [MeSH Terms] |
| #14 | limb function [Title/Abstract] |
| #15 | muscle function [Title/Abstract] |
| #16 | motor function [Title/Abstract] |
| #17 | exercise Function [Title/Abstract] |
| #18 | balance [Title/Abstract] |
| #19 | postural control [Title/Abstract] |
| #20 | balance control [Title/Abstract] |
| #21 | physical performance [Title/Abstract] |
| #22 | #6 OR #7 OR #8 OR #9 OR #10 #11 OR #12 OR #13 OR #14 OR #15 OR #16 OR #17 OR #18 OR #19 OR #20 OR #21 |

| | |
|---|---|
| #23 | cognition [MeSH Terms] |
| #24 | cognitive function [MeSH Terms] |
| #25 | cognition [Title/Abstract] |
| #26 | cognitive function [Title/Abstract] |
| #27 | #23 OR #24 OR #25 OR #26 |
| #28 | frail elderly [MeSH Terms] |
| #29 | frail older adults [MeSH Terms] |
| #30 | sarcopenia [MeSH Terms] |
| #31 | frail elderly [Title/Abstract] |
| #32 | frail older adults [Title/Abstract] |
| #33 | sarcopenia [Title/Abstract] |
| #34 | #28 OR #29 OR #30 OR #31 OR #32 OR #33 |

The search strategy will be modified as required for other electronic databases.
